# Reshaping the research landscape in Brazil

**DOI:** 10.7554/eLife.90533

**Published:** 2023-07-05

**Authors:** Gustavo Schiavone Crestana, Jéssica Mendes, Renato Augusto Corrêa dos Santos, Flávia Vischi Winck

**Affiliations:** 1 Luiz de Queiroz College of Agriculture (ESALQ), University of São Paulo Piracicaba Brazil; 2 Center of Nuclear Energy in Agriculture (CENA), University of São Paulo Piracicaba Brazil

**Keywords:** point of view, Brazil, science policy, funding, careers in science, early-career researchers, science, politics, None

## Abstract

Brazil would benefit from a long-term strategy for science and innovation that improves the standing of both science and scientists in the country.

The past few years have been tough for science in Brazil. Jair Bolsonaro, who was president from 2019–2022, and his government were viewed by most scientists as anti-science. For example, the Bolsonaro administration has consistently rejected the scientific consensus on climate change and did not support vaccination at the start of the COVID-19 pandemic. The spread of misinformation about scientific topics was exacerbated by the popularity of social media (Diele-Viegas et al., 2021). The federal budget for science and education has also fallen over the past decade, from around 17% of the total budget in 2013 to about 8% in 2020 (OECD, 2021). Graduate students and early-career researchers were hit particularly hard by these cuts. Indeed, in the last month of Bolsonaro’s tenure, some students were not able to pay their rent or buy food (CAPES, 2022).

The National Innovation System (NIS) in Brazil consists of a range of public and private institutions, including universities, research institutes, industry associations, and government agencies (Figure 1; Mazzucato and Penna, 2016). These institutions work together to support research and development, technology transfer, and the commercialization of new products and services. The National Innovation System is crucial for researchers because it supports research projects and funds scholarships at universities and research institutes across Brazil. However, both these areas were impacted by the budget cuts of the past decade.

**Figure 1.**
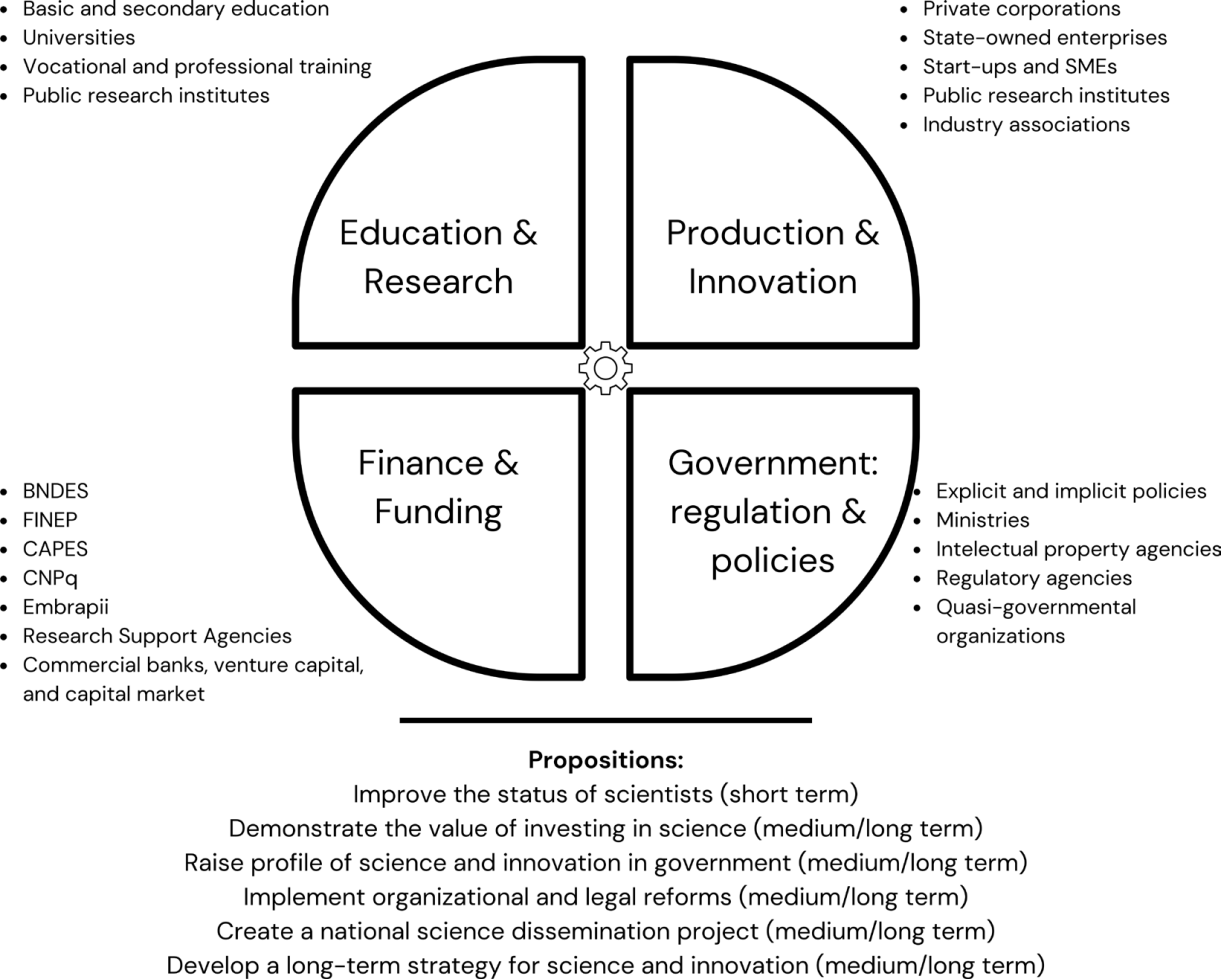
Reforming science and innovation in Brazil. The National Innovation System (NIS) in Brazil consists of a range of public and private institutions (including universities, research institutes, industry associations, and government agencies) that work together to support research and development, technology transfer, and the commercialization of new products and services. The four elements of the system are: education and research; production and innovation; finance and funding; and government (which is responsible for regulation and policy). The six propositions for reforming the system are described in the main text.

Adapted from Mazzucato and Penna, 2016.

Luiz Inácio Lula da Silva (also known as Lula), who was President between 2003 and 2010, defeated Bolsonaro in the presidential election last October and took over in January of this year. During his previous time as President, Lula implemented a number of policies that supported science and education in Brazil. This time around his government has announced plans to restructure the Ministry of Science, Technology and Innovation and the agencies that fund science, such as the CNPq (short for the National Council for Scientific and Technological Development) and the CAPES Foundation (Coordination for the Improvement of Higher Education).

The new administration has also raised pay for researchers on federal scholarships and announced that it plans to increase the number of scholarships available. This is clearly good news, but there are still reasons to be worried about the future of science in Brazil. One overarching problem is the lack of a long-term strategy for scientific research and innovation, including a target for investments in science and education as a percentage of gross domestic product (GDP). In this article we summarize six specific problems and challenges confronting research and innovation in Brazil, and make recommendations on how to address them.

First, low salaries, poor working conditions and a lack of benefits are driving many young researchers out of science and/or out of Brazil. There is an urgent need to increase salaries and improve working conditions and benefits, especially for researchers at the start of their careers. Ideally, being a scientific researcher at any level or stage would be officially recognized by the government as a profession – similar to engineers, physicians and lawyers – which would help scientific researchers have rights such as vacation, retirement plans and pensions, complementary health care, and paid maternity and paternity leave.

The second challenge is to demonstrate to the Brazilian population that investing in science and innovation will lead to discoveries and new knowledge that will improve people’s lives and address many of the challenges facing society today. One way to do this would be to use an approach called the Social Return of Investment (SROI) that is employed by various non-profit organizations and government agencies to assess the social, environmental and economic impacts of projects they fund. For example, a study that looked at the impact of scholarships, research projects and infrastructure funded by the São Paulo Research Foundation in the fields of agronomy and agriculture showed that a return of R$ 27 was generated for every R$ 1 invested in the state of São Paulo (De Araújo and Nicolella, 2016). We suggest that similar studies be undertaken to assess the impact of the money spent on science by state and federal agencies in Brazil.

The third challenge is to ensure that science and innovation has a higher profile in the Brazilian parliament and in government departments. For this to happen, we need to encourage the development of future generations of leaders who are more receptive to dialogue and open to the possibilities that science offers. The proposal to set up a Parliamentary Front for Science, Technology, Research, and Innovation in the Chamber of Deputies (the lower house of the National Congress of Brazil) is an encouraging step in the right direction: this body would, among other things, seek to modernize the National Innovation System and to encourage research and innovation in areas that are of strategic importance to the country. There might also be a role for non-profit organizations such as RenovaBr, a non-profit that aims to train political leaders in Brazil, and has a good record of promoting the use of science and evidence in policy making.

The fourth challenge involves various organizational and legal reforms. These should include enhancing institutional autonomy (and reducing bureaucracy) and making it easier for public and private organizations to collaborate. With the exception of agroindustry (Cassiolato, 2007), there have been relatively few collaborations between public bodies (such as universities and research institutes) and private companies that have resulted in impactful technological development in Brazil (Cassiolato, 2015). There is also a need for the various institutions in the National Innovation System to take account of a new legal framework called the MLCTI in their programs, projects and calls for proposals. The MLCTI is a set of laws, regulations and guidelines that establish the legal framework for the development of scientific research, technology, and innovation in Brazil. It has brought about several important changes, such as greater flexibility in the rules for hiring researchers, the simplification of processes for importing equipment and supplies for research, and the creation of tax incentives for companies investing in research, development and innovation. State laws about innovation will also need to be updated to take account of MLCTI.

The fifth challenge is to facilitate meaningful engagement between research institutions and society in order to combat the current wave of scientific denialism in Brazil. A number of organizations are already doing good work in this area – notably the Institut Questão de Ciência (IQC) and the Bori Agency – but there is a need to do more. One option would be to create a national project for science dissemination, and to increase the number of teaching and research positions related to these areas at universities and research institutes.

The challenges discussed so far are all interconnected, which makes clear the need for a long-term strategy for science and innovation in Brazil. Agriculture has been a success story in Brazil in recent decades (Correa and Schmidt, 2014), and the Brazilian Agricultural Research Corporation (Embrapa) has been one of the driving forces behind this. A working group has been set up to review the national agricultural research system, of which Embrapa is a crucial part, and to plan for the future in response to both local and global challenges and opportunities. A similar approach needs to be taken with the National Innovation System.

The challenges faced by science in Brazil over the past few years have been significant. The anti-science stance of the previous administration, coupled with budget cuts and a lack of support for research and education, has had a detrimental impact on the scientific community, particularly graduate students and early-career researchers. However, the recent change of government and the implementation of new policies offer a glimmer of hope for the future. The restructuring of institutions and increased funding for research and scholarships, in particular, demonstrate a commitment to prioritize science and innovation. Nonetheless, as we have discussed here, there are still obstacles to overcome and we need to act now if we want to ensure a prosperous future for science in Brazil.

## Data Availability

There are no data associated with this article.
